# *Ex-Vivo* Uterine Environment (EVE) Therapy Induced Limited Fetal Inflammation in a Premature Lamb Model

**DOI:** 10.1371/journal.pone.0140701

**Published:** 2015-10-16

**Authors:** Yuichiro Miura, Masatoshi Saito, Haruo Usuda, Eleanor Woodward, Judith Rittenschober-Böhm, Paranthaman S. Kannan, Gabrielle C. Musk, Tadashi Matsuda, John P. Newnham, Matthew W. Kemp

**Affiliations:** 1 School of Women's and Infants' Health, The University of Western Australia, Crawley, Western Australia, Australia; 2 Center for Perinatal and Neonatal Medicine, Tohoku University Hospital, Sendai, Miyagi, Japan; 3 Division of Neonatology, Pediatric Intensive Care and Neuropediatrics, Medical University of Vienna, Vienna, Austria; 4 Division of Pulmonary Biology, Cincinnati Children's Hospital Medical Center, Cincinnati, Ohio, United States of America; 5 School of Veterinary and Life Sciences, Murdoch University, Murdoch, Western Australia, Australia; 6 Animal Care Services, The University of Western Australia, Crawley, Western Australia, Australia; Hospital de Especialidades del Niño y la Mujer de Queretaro, MEXICO

## Abstract

**Introduction:**

*Ex-vivo* uterine environment (EVE) therapy uses an artificial placenta to provide gas exchange and nutrient delivery to a fetus submerged in an amniotic fluid bath. Development of EVE may allow us to treat very premature neonates without mechanical ventilation. Meanwhile, elevations in fetal inflammation are associated with adverse neonatal outcomes. In the present study, we analysed fetal survival, inflammation and pulmonary maturation in preterm lambs maintained on EVE therapy using a parallelised umbilical circuit system with a low priming volume.

**Methods:**

Ewes underwent surgical delivery at 115 days of gestation (term is 150 days), and fetuses were transferred to EVE therapy (EVE group; n = 5). Physiological parameters were continuously monitored; fetal blood samples were intermittently obtained to assess wellbeing and targeted to reference range values for 2 days. Age-matched animals (Control group; n = 6) were surgically delivered at 117 days of gestation. Fetal blood and tissue samples were analysed and compared between the two groups.

**Results:**

Fetal survival time in the EVE group was 27.0 ± 15.5 (group mean ± SD) hours. Only one fetus completed the pre-determined study period with optimal physiological parameters, while the other 4 animals demonstrated physiological deterioration or death prior to the pre-determined study end point. Significant elevations (*p*<0.05) in: **i)** inflammatory proteins in fetal plasma; **ii)** selected cytokine/chemokine mRNA expression levels in fetal tissues; and **iii)** histological inflammatory score in fetal lung, were observed in the EVE group compared to the Control group. There was no significant difference (*p*>0.05) in surfactant protein mRNA expression level between the two groups.

**Conclusion:**

In this study, we achieved limited fetal survival using EVE therapy. Despite this, EVE therapy only induced a modest fetal inflammatory response and did not promote lung maturation. These data provide additional insight into markers of treatment efficacy for the assessment of future studies.

## Introduction

Preterm birth (live delivery prior to 37 weeks’ gestation) is responsible for at least 1 million perinatal deaths every year [1]. Despite significant advances in perinatal medicine (i.e. antenatal corticosteroid administration, surfactant therapy, advanced ventilation strategies), a significant proportion of infants born between 22 and 25 weeks’ gestation in high-resource settings will either die or experience life-long diseases of the cardiovascular, respiratory or neurosensory systems [2,3]. It is unclear if the high incidence of morbidity and mortality in this population is due to the antenatal exposures responsible for prematurity, or is as a result of forcibly adapting the highly immature physiology of a 22–25 week gestation fetus to pulmonary gas exchange in *ex-uterine* life. We have speculated that treating very premature babies without mechanical ventilation may allow them to be supported without causing or exacerbating injury. Designing treatment strategies that take advantage of the fetal-like physiology exhibited by extremely preterm infants may decrease morbidity and mortality of early preterm babies who cannot survive with existing neonatal intensive care.

*Ex-vivo* uterine environment (EVE) therapy is a novel treatment strategy based on the concepts introduced above. It uses an artificial placenta to provide extracorporeal gas exchange and nutrient delivery to a fetus submerged in a warmed amniotic fluid bath. An artificial placenta is a system that allows for arterio-venous extracorporeal life support using the umbilical vasculature. Previously, we demonstrated the efficacy of our EVE system with the pumpless artificial placenta using the preterm lamb model [4].

Systemic fetal inflammation, characterised in humans as the fetal inflammatory response syndrome (cord blood interleukin-6 concentration >11 pg/mL [5]) is strongly associated with fetal injury, in particular brain injury, in the setting of preterm birth. Thus, any treatments for extremely preterm infants, such as EVE therapy, must allow for the control of pathological fetal inflammation. Intrauterine inflammation is also associated with precocious maturation of the fetal lung [6,7]. It has previously been reported that extracorporeal life support causes systemic inflammation [8–14], and that the intensity of inflammation is altered by the blood contact area [15] and the coating of the surface area [16]. As such, the inflammatory responses generated by fetuses maintained on EVE therapy are of significant interest. In this study, the systemic inflammatory response of preterm lambs undergoing EVE therapy was assessed.

Our primary hypothesis was that, when successfully functioning, the EVE system in this study would cause minimal inflammation to the fetuses as it incorporated a bespoke, low-volume membranous oxygenator with a heparin coating. On the other hand, we also hypothesised that systemic inflammatory changes would become apparent in animals in which treatment was unsuccessful. As such, changes in fetal inflammatory status may serve as a useful means of assessing treatment success in real-time. Based on our primary hypothesis, we also anticipated that successful adaptation to EVE therapy would not induce fetal lung maturation. To test these hypotheses, experiments were conducted using a premature lamb model. The primary aim was to compare the concentration of inflammatory/stress markers in fetal plasma and the expression of inflammatory cytokine/chemokine mRNA in fetal tissues between the EVE treatment and the Control groups, and also between successful and unsuccessful cases in the EVE treatment group. Our secondary aim was to assess the expression of surfactant protein (SP) mRNA in fetal lung between the EVE treatment and the Control groups.

## Materials and Methods

### Experimental protocol

All procedures involving animals were performed in Perth, Western Australia following review and approval by the Animal Ethics Committee of the University of Western Australia (approval RA/3/100/1289). All surgery was performed under the anaesthesia with isoflurane, and all efforts were made to minimise suffering.

### Study group

#### a) EVE group

5 *merino-cross ewes* with timed, singleton pregnancies underwent surgery at 115 days gestational age (dGA; term = 150). Animals were fasted for 12 hours before surgery with *ad libitum* access to water. Ewes were anaesthetised, intubated, and ventilated during the surgical procedure. Intravenous fluids (0.9% NaCl) were administered at a rate of 10 mL·kg^-1^·hour^-1^. The ewe’s abdomen was clipped to expose the skin and thoroughly prepared for surgery with as previously described [17]. After a maternal laparotomy and hysterotomy, catheters were placed in fetal jugular vein (4 French, Arrow Pediatric two-lumen central venous catheter, Teleflex Medical, Morrisville, NC), carotid artery (4.5 French, PediaSat Oximetry Catheter, Edwards Lifesciences, Irvine, CA), two umbilical arteries (8 French, Fem-Flex II; Edwards Lifesciences), and one umbilical vein (10 French, Argyle Trocar catheter; Covidien, Dublin, Ireland). The fetus was connected to the artificial placenta, and then delivered and carefully submerged in an amniotic fluid bath. The fetuses were continuously treated with heparin (12.5 U·kg^-1^·hour^-1^) to prevent blood coagulation and prostaglandin E_1_ (50 ng·kg^-1^·min^-1^) to prevent closure of the *ductus arteriosus*; and periodically treated with cefazolin (40 mg·kg^-1^·day^-1^) to prevent infection. Activated clotting times were monitored using a Hemochron Signature Elite (Accriva Diagnostics, San Diego, CA) and maintained at >180 seconds. Appropriate nutrient supplementations including glucose (10–12%), amino acids (1.0–1.2 g·kg^-1^·day^-1^), and lipid (0.7–1.0 g·kg^-1^·day^-1^) were delivered via the catheterised fetal jugular vein. Fetuses were constantly monitored to ensure that adequate sedation was maintained, and intravenous bolus of midazolam (0.2 mg/kg) was administered when needed. Each fetus was supposed to maintain in the EVE system for pre-determined 40 hours.

#### b) Control group

6 ewes with timed, singleton pregnancies were surgically delivered at 117 dGA. Fetuses were cannulated and blood samples collected at the time of surgical delivery under non-recovery anaesthesia. Fetuses were then immediately euthanised to allow for tissue sample collection.

All ewes were euthanised with an intravenous bolus of pentobarbitone (160 mg/kg) at the time of fetal delivery. All fetuses in the Control group were euthanised with an intravenous pentobarbitone (100 mg/kg) at 117 dGA. All fetuses in the EVE group were euthanised with an intravenous pentobarbitone (100 mg/kg) at the pre-determined time point (n = 2) or acutely following irreversible deterioration of key physiological variables (either total circuit blood flow<20 mL·kg^-1^·min^-1^ or SaO_2_<20% for more than 10 minutes; n = 3).

### EVE Components

#### a) Artificial Placenta

The circuit was composed of three main components: **i)** outflow tubes; **ii)** membranous oxygenators; and **iii)** an inflow tube. Two membranous oxygenators were placed in a parallel orientation. Heparinised polyvinyl chloride tubes were used for both the inflow and outflow tubes. The priming volume of whole circuit was 70 mL, and the circuit was primed with heparinised maternal blood [18]. Total membrane surface area was 0.3 m^2^. Circuit flow was maintained by the fetal heart. Appropriate oxygen was supplied to the membranous oxygenators to keep fetal PaO_2_ between 10–30 mm Hg.

#### b) Amniotic fluid bath

Both the fetus and the artificial placenta circuit were submerged in a sealed bath of artificial amniotic fluid held at a constant 38.9°C. pH and electrolyte concentration of artificial amniotic fluid were identical to that of amniotic fluid in merino sheep (pH: 7.2 ± 0.2, Na^+^: 122 ± 10 mEq/L, Cl^-^: 112 ± 10 mEq/L, all values represent group mean ± standard deviation (SD)). Amniotic fluid was constantly filtered and UV (isolation) irradiated to prevent microbial colonisation.

### Data Acquisition

#### Data relating to the physiological parameters

Fetal heart rate and mean arterial pressure were continuously monitored and recorded using a SurgiVet (Smiths Medical, St. Paul, MN). Fetal arterial oxygen saturation was continuously monitored and recorded using a Vigileo Monitor (Edwards Lifesciences). Circuit blood flow (mL/min) was continuously monitored using electromagnetic flow sensors (Transonic 400-Series, Transonic Systems Inc., Ithaca, NY) which were attached to the arterial positions of the blood circuit, and recorded using a PowerLab (ADInstruments, Dunedin, New Zealand). pH, PaO_2_, PaCO_2_, blood lactate level (Siemens Rapidlab 1200, Siemens, Munich, Germany), and activated clotting time were measured in blood samples obtained from the fetal carotid artery at least every 4 hours. Blood gas data were corrected using fetal core temperature. Fetal arterial blood for complete blood counts was collected in a 10 mL Vacutainer (Becton, Dickinson and Company, Franklin Lakes, NJ) containing EDTA. Haematological analyses were performed by an independent pathology laboratory (Vetpath, Perth, WA, Australia).

#### Data relating to the assessment of inflammation

Fetal arterial blood for inflammatory cytokine/chemokine mRNA analyses was intermittently collected in a PAXgene blood RNA tube (PreAnalytiX, Hombrechtikon, Switzerland). Fetal plasma samples were also intermittently collected for tumour necrosis factor (TNF) –α and monocyte chemo-attractant protein (MCP) -1 protein analyses. Fetal arterial blood for liver function tests was collected in a 10 mL SST clot-activating Vacutainer (Becton, Dickinson and Company) at the end of protocol. Biochemical analyses were performed by Vetpath (Perth, WA, Australia).

Fetal tissues (lung right lower lobe, axilla skin, liver, and cerebral cortex) for inflammatory cytokine/chemokine mRNA analyses were collected and snap-frozen in liquid nitrogen at the end of the protocol. Fetal lung (right upper lobe) was also collected at the end of the protocol, and inflation-fixed in 10% neutral buffered formalin using constant pressure (30 cm H_2_O) for 24 hours before being washed in phosphate buffered saline and embedded in paraffin.

#### Data relating to the assessment of pulmonary maturation

Fetal lung right lower lobe for surfactant protein mRNA analyses was collected and snap-frozen in liquid nitrogen at the end of the protocol.

### Post processing section

Fetal tissues were homogenised at 6,500 rpm, 15 seconds (brain), 30 seconds (lung and liver); or 20 seconds, 2 cycles (skin) using a Precellys 24 macerator (Bertin Technologies, Montigny-le-Bretonneux, France). Total RNA was extracted using TRIzol (Life Technologies, Carlsbad, CA). Extracted RNA was treated with Turbo-DNase (Life Technologies) in accordance with manufacturer’s instructions to remove any residual DNA. RNA template was quantified using a Qubit 2.0 Fluorometer (Life Technologies) using a broad-range RNA quantitation kit (Life Technologies). RNA extracts were diluted in nuclease-free water (Life Technologies) to a final concentration of 25 ng/μL.

Total RNA was extracted from immunocytes in fetal whole blood using PAXgene Blood RNA Kit (PreAnalytiX) in accordance with manufacturer’s instructions. RNA quantification and dilution were demonstrated in the same way described above.

Ovine-specific PCR primers and hydrolysis probes for SP-A, B, C, D, interleukin (IL)-1β, IL-6, IL-8, TNF-α, MCP-2, C-reactive protein (CRP), Serum amyloid A (SAA) 3, and Hepcidin (all Life Technologies) were used to perform quantitative PCR. Reactions were performed using an EXPRESS One-Step SuperScript qRT-PCR Kit (Life Technologies) with 125 ng of template fetal tissue RNA in a total volume of 20 μL as per manufacturer’s instructions. Reaction cycling conditions were: 15 minutes reverse transcription at 50°C and an initial denaturation/polymerase activation at 95°C for 20 seconds, followed by 40 cycles of 95°C for 3 seconds and 60°C for 30 seconds (data acquisition phase). All reactions were performed in the fast 96-well plates on a ViiA7 real-time PCR thermocycler (Life Technologies). Target Cq values were normalised to 18S rRNA Cq value and expressed as fold changes relative to pooled control values. Reaction efficiencies were within limits proposed in the MIQE guidelines [19]. dCq values were used to perform statistical analyses for significant differences between the Control group *vs*. the EVE group.

Quantification of TNF-α and MCP-1 protein concentrations in fetal plasma samples was performed using a commercial kit from Kingfisher Biotech (St. Paul, MN) with washing performed on a Biosan platewasher (3D-IW8, Inteliwasher, Biosan, Riga, Latvia). Standards (calibration curve R^2^>0.99) were assayed in triplicate (average coefficient of variation 7.8%) and unknown samples were assayed in duplicate. The assay limit of detection was <4 pg/mL. 100 μL of each standard or sample was incubated overnight (16 hours) at 4°C. The remainder of the assay was performed in accordance with the manufacturer’s instructions, with absorbance at 450 nm read on an Anthos 2010 Microplate Reader (Biochrom Ltd., Cambridge, United Kingdom).

**Fig 1:**
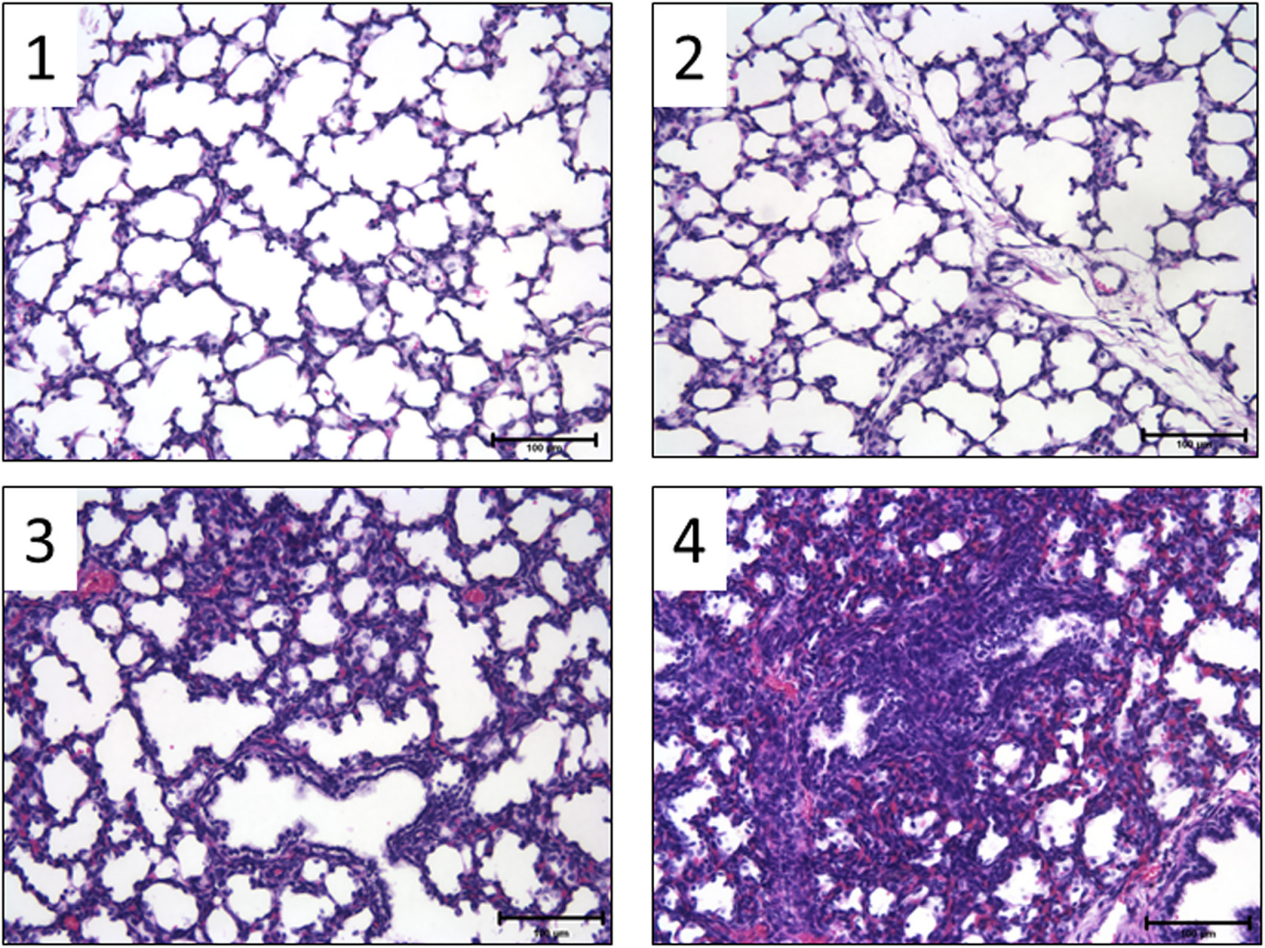
Representative images for qualitative scoring of airspace infiltration. Images are representative of the indicated inflammatory scores assigned to each field assessed (× 200 total magnification). Scale bar = 100 μm.

5 μm paraffin sections of 10% (pH 7.4) formalin-fixed fetal lung (right upper lobe) were stained with Meyer’s haematoxylin and eosin. Qualitative scoring of airspace infiltration was performed by a single investigator blinded to treatment groups. Six fields (200 x total magnification) were scored for each animal. Airspace infiltration and consolidation were graded as follows: 0 = no inflammatory cells in airspace; 1 = airspace inflammatory cells, no consolidation; 2 = airspace inflammatory cells + limited microconsolidation (1–4 per field) foci; 3 = airspace inflammatory cells + numerous (>4 per field) but predominantly discrete microconsolidation foci; and 4 = airspace inflammatory cells + confluent airspace consolidation (Fig 1).

### Statistical Analysis

Normally distributed values were expressed as the group mean ± SD, while non-parametric values were expressed as the group median [interquartile range (IQR)]. Analyses were performed using IBM SPSS for Windows, Version 20.0 (IBM Corporation, Armonk, NY). Data were tested for normality using Shapiro-Wilk test. In the comparison of two groups, for normally distributed data, mean differences were tested for significance using *t*-test. Between-group differences in non-parametric data were tested for significance using Mann-Whitney U test. In the comparison of more than three groups, for normally distributed data, mean differences were tested for significance using one-way ANOVA. Multiple *post-hoc* comparisons were performed using Tukey’s test. Between-group differences in non-parametric data were tested for significance using Kruskal-Wallis one-way ANOVA. Multiple *post-hoc* comparisons were performed using the rank sum test. All *p* values<0.05 were accepted as significant.

## Results

### Data relating to the physiological parameters

Table 1 shows the comparison between the Control and the EVE groups. There were no unexpected deaths in the Control group. There was no statistically significant difference in any delivery data shown in Table 1 between the two groups.

**Table 1:** The comparison between the Control and the EVE groups.

	Control	EVE	Statistical test	p value
Number	6	5		
Sex (m/f)	3/3	4/1	Chi-Square test	0.30
Survival time (hours)	N/A	27.0 ± 15.5		
Body Weight (g)	2097 ± 194	2209 ± 124	t-test	0.29
RBC (×10^12^/L)	7.3 ± 1.0	9.4 ± 3.7	t-test	0.20
Haemoglobin (g/dL)	11.5 ± 1.5	13.2 ± 5.4	t-test	0.48
WBC (×10^9^/L)	2.5 ± 1.4	3.9 ± 2.4	t-test	0.28
AST (U/L)	26.2 ± 4.4	90.8 ± 101.9	t-test	0.29
GLDH (U/L)	3.7 [11.8]	17.5 [771.4]	Mann-Whitney U test	0.14
Total Bilirubin (μmol/L)	10.5 [2.3]	10.5 [52.0]	Mann-Whitney U test	0.91
Albumin (g/L)	20.7 ± 2.3	15.8 ± 9.2	t-test	0.24

AST, aspartate aminotransferase; GLDH, glutamate dehydrogenase.

Normally distributed values are expressed as the group mean ± SD, while non-parametric values are expressed as the group median [IQR]. Significant difference was observed in no parameter between the two groups.

**Fig 2:**
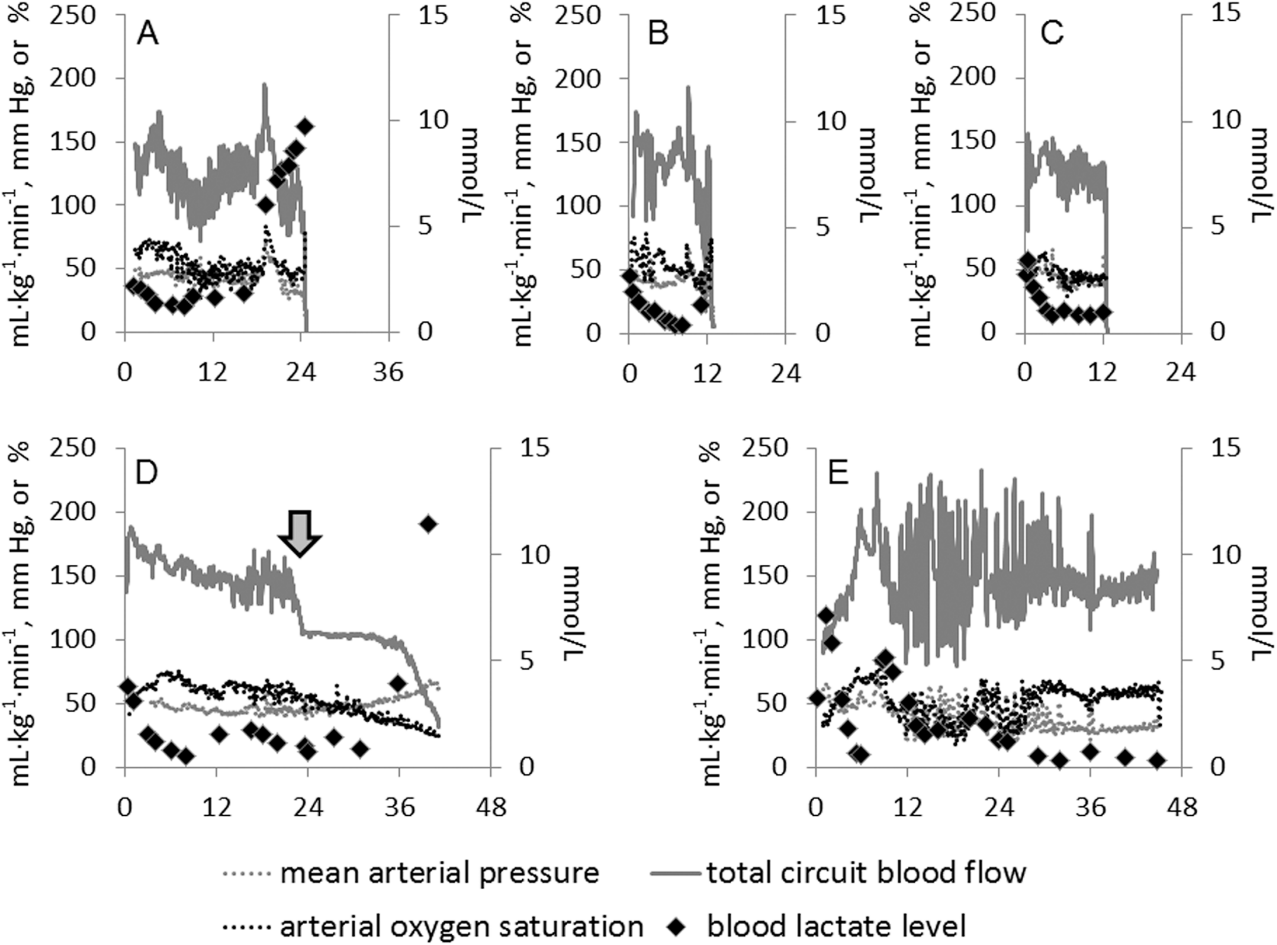
Changes in fetal parameters over time in EVE group. The horizontal axes represent the time after induction of EVE therapy (hours). The grey dotted lines show mean arterial pressure (mm Hg), the grey solid lines show total circuit blood flow (mL·kg^-1^·min^-1^), the black dotted lines show arterial oxygen saturation (%), and the diamonds show blood lactate level (mmol/L). Only the blood lactate levels use the right scale bars. Individual case information is as follows: **A)** The blood lactate level started to elevate at 19 hours, and continued increasing although other parameters kept stable. Congestive heart failure resulted in a sudden drop of circuit blood flow at 24 hours and euthanasia. **B)** All the parameters remained within the reference range after stabilisation; however the circuit corrupted (embolism of artificial amniotic fluid) at 12.5 hours, resulting in euthanasia. **C)** All the parameters remained within the reference range before sudden fatal arrhythmia occurred at 12.5 hours, resulting in euthanasia. **D)** One membranous oxygenator was blocked with a blood clot at 23 hours (arrow). Despite gradual fetal deterioration including increased the blood lactate levels and decreased circuit blood flow, the fetus completed the pre-determined 40-hour study period. **E)** Although some fluctuations of parameters were observed in the former period, the fetus completed the pre-determined 40-hour study period with stable physiological parameters.

Fig 2 shows changes in mean arterial pressure, circuit blood flow, arterial oxygen saturation, and blood lactate levels after induction of EVE therapy from fetuses euthanised prior to 40 hours EVE therapy due to irreversible deterioration of circuit blood flow (Fig 2A–2C) and fetuses that completed pre-determined 40 hours under EVE therapy (Fig 2D and 2E).

### Data relating to the assessment of inflammation

**Fig 3:**
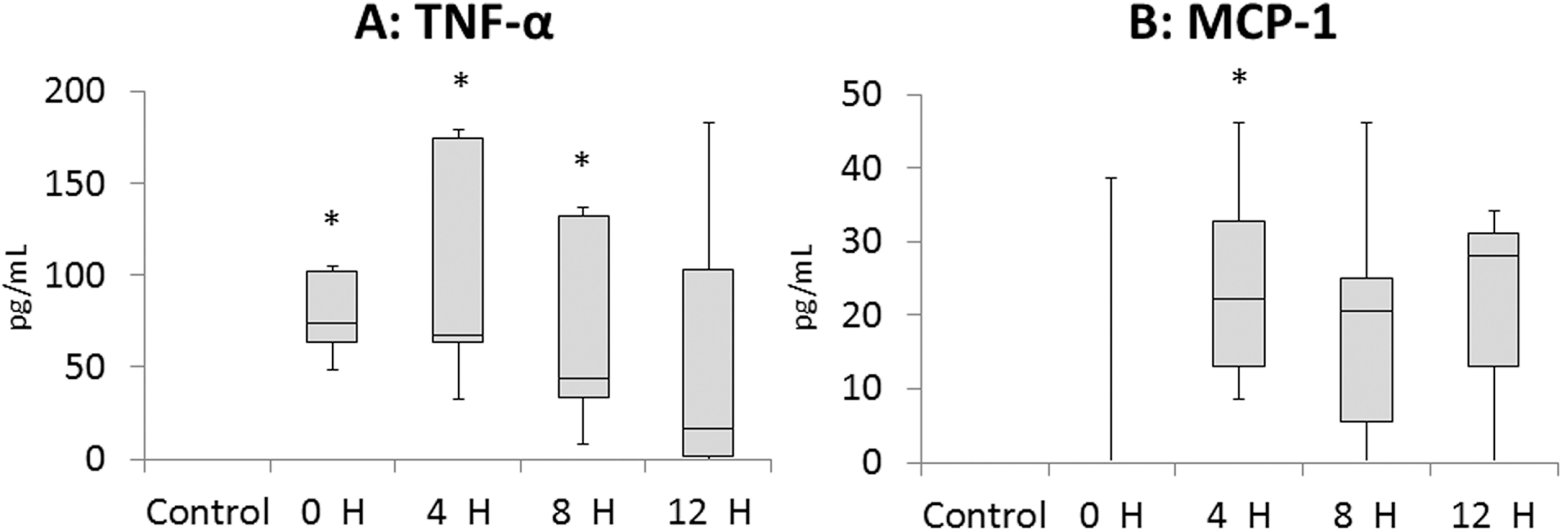
Inflammatory protein concentration in fetal plasma measured with ELISA. H: hours after induction of EVE therapy. Data are presented as box plots with the group median, with whiskers representing maximum and minimum values. Fig 3A shows the data for TNF-α and Fig 3B shows the data for MCP-1. Significant differences *vs*. value for Control group are indicated: *, *p*<0.05.

The TNF-α protein concentration in fetal plasma immediately, 4 hours, and 8 hours after the induction of EVE therapy were significantly higher than that of Control group (Fig 3A). Meanwhile, the MCP-1 protein concentration in fetal plasma 4 hours after the induction of EVE therapy was significantly higher than that of Control group. No significant difference was detected between the Control and the EVE groups at subsequent time points assessed (Fig 3B).

**Fig 4:**
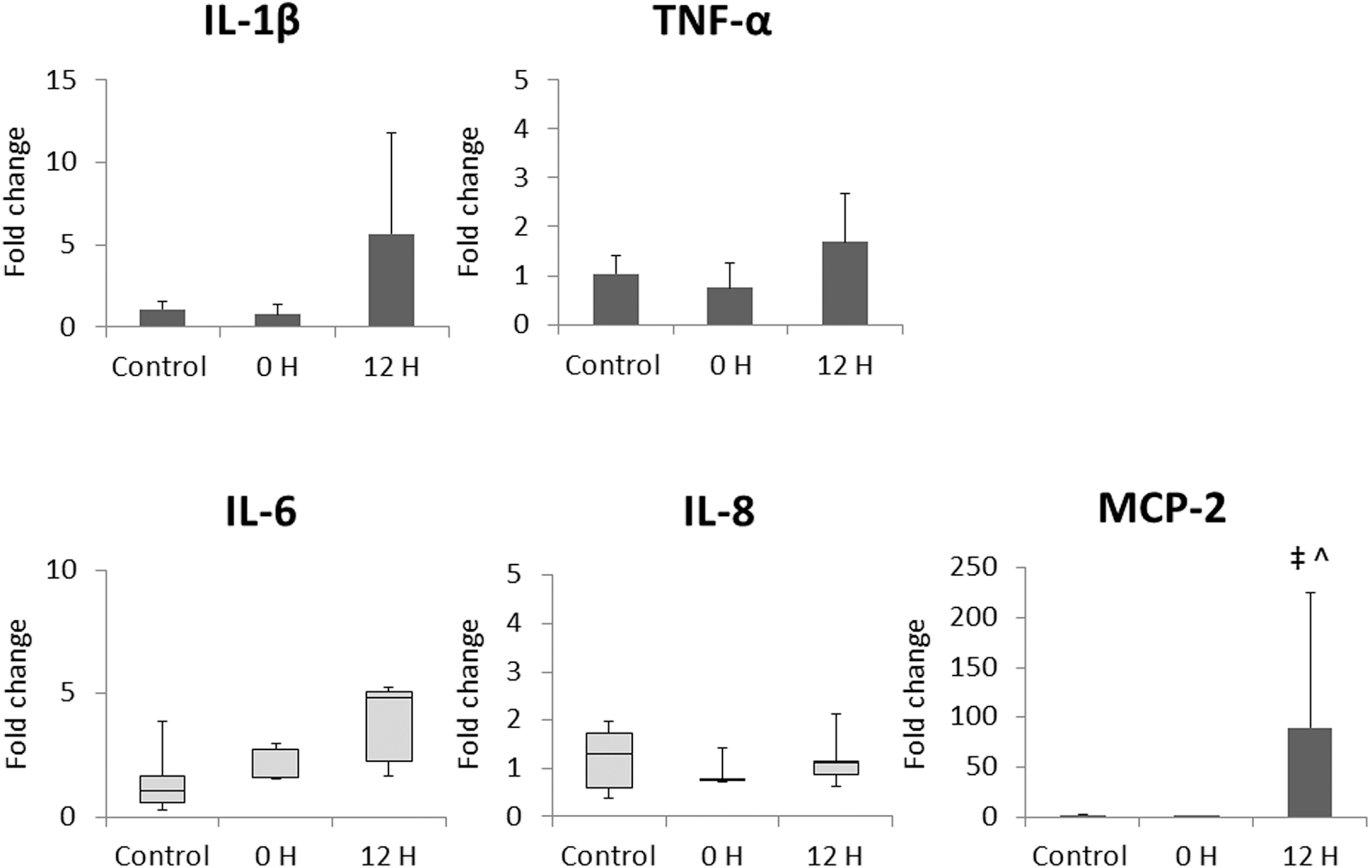
Relative expression of inflammatory cytokine/chemokine mRNA levels in fetal circulating immunocytes measured with quantitative PCR. H: hours after induction of EVE therapy. Values of IL-1β, TNF-α, and MCP-2 are presented as bar charts with the group mean normalised expression *vs*. the value for Control group, with error bars representing SD. Values of IL-6 and IL-8 are presented as box plots with the group median normalised expression *vs*. the value for Control group, with whiskers representing maximum and minimum values. Significant difference *vs*. value for Control group is indicated: ^‡^, *p*<0.01; significant difference *vs*. value for 0 H group is indicated: ^, *p*<0.01.

Significant elevation in MCP-2 mRNA expression level in circulating immunocytes isolated from fetal arterial blood was detected at 12 hours after induction of EVE therapy compared to both the Control and the 0 hour point in the EVE group (Fig 4).

**Fig 5:**
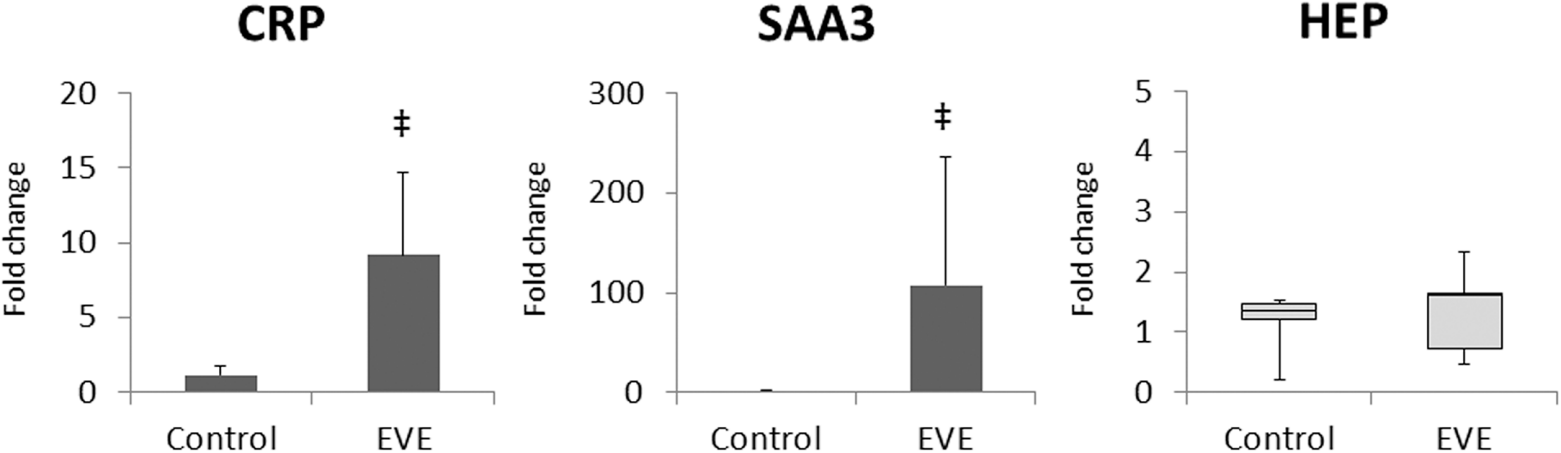
Relative expression of fetal liver acute phase protein mRNA levels measured with quantitative PCR. CRP, C-reactive protein; SAA3, Serum amyloid A 3; HEP, Hepcidin. Values of CRP and SAA3 are presented as bar charts with the group mean normalised expression *vs*. the value for Control group, with error bars representing SD. Values of HEP are presented as box plots with the group median normalised expression *vs*. the value for Control group, with whiskers representing maximum and minimum values. Significant differences *vs*. value for Control group are indicated: ^‡^, *p*<0.01.

Significant elevations in CRP and SAA3 mRNA expression levels in the fetal liver were detected in the EVE group compared to the Control group (Fig 5).

**Fig 6:**
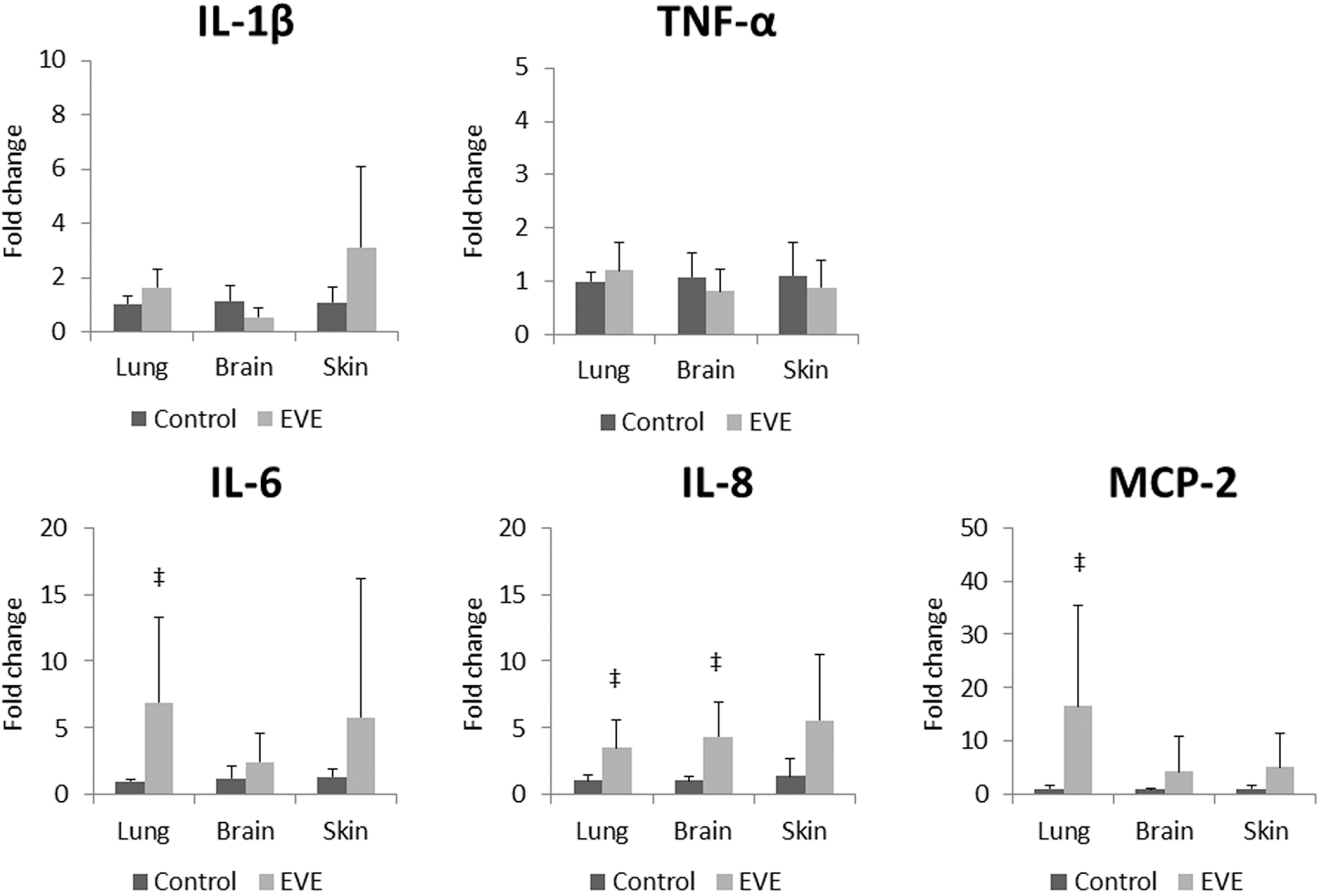
Relative expression of inflammatory cytokine/chemokine mRNA levels in fetal tissues measured with quantitative PCR. All values are presented as bar charts with the group mean normalised expression *vs*. the value for Control group, with error bars representing SD. Significant differences *vs*. value for Control group are indicated: ^‡^, *p*<0.01.

Significant elevations in IL-6, IL-8, and MCP-2 mRNA expression levels in the fetal lung; IL-8 mRNA expression level in the fetal brain, were detected in the EVE group compared to the Control group. Meanwhile, no significant difference was observed in any fetal skin mRNA expression level between the two groups (Fig 6).

**Fig 7:**
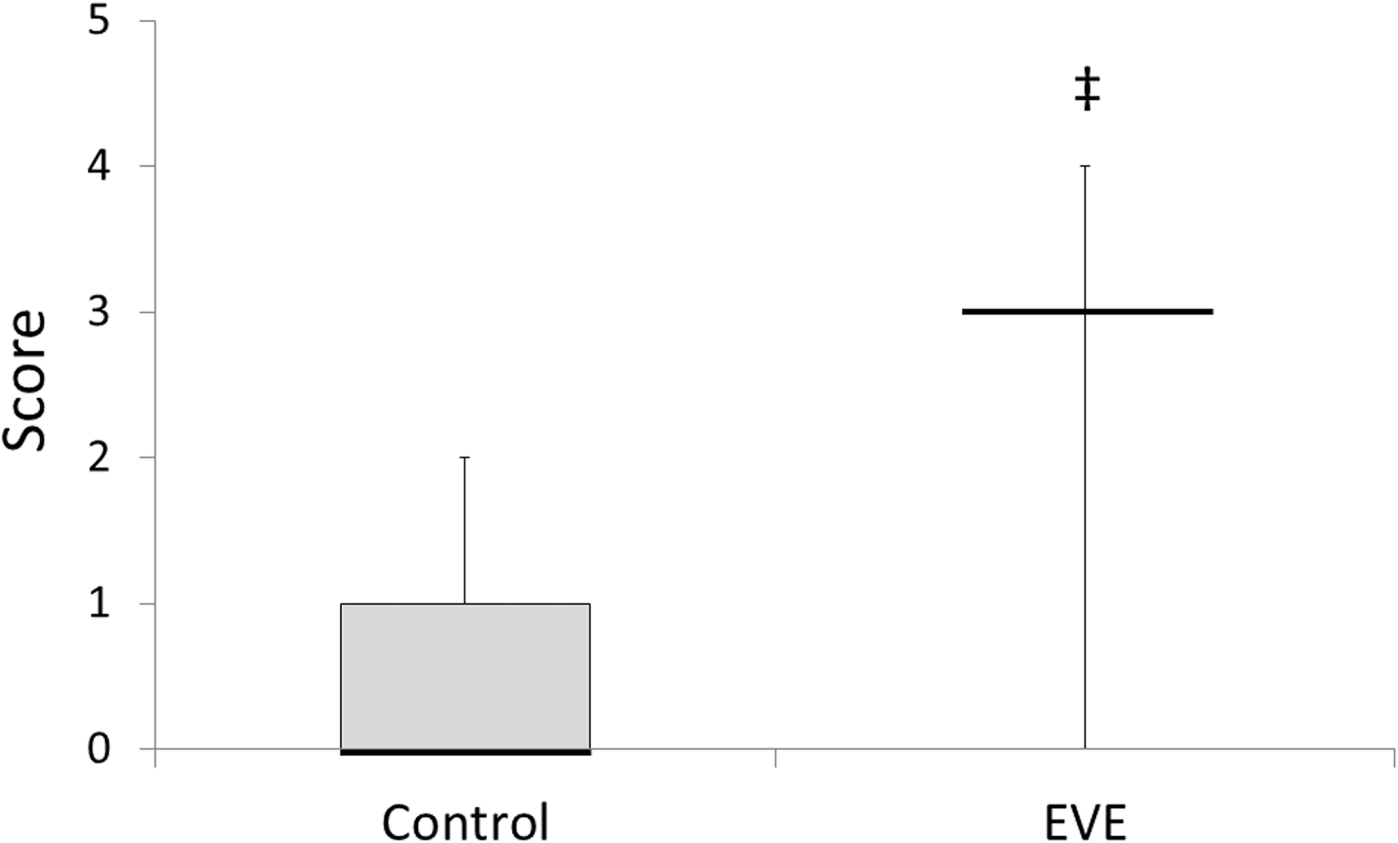
Qualitative scoring of histological inflammation in fetal airspace. Data are presented as box plots with the group median, with whiskers representing maximum and minimum values. Significant difference *vs*. value for Control group is indicated: ^‡^, *p*<0.01.

Significant increase in inflammatory score in fetal airspace was detected in the EVE group compared to the Control group (Fig 7).

Among five fetuses in the EVE group, there were no apparent differences in systemic or tissue inflammatory markers between the fetus with stable parameters (case E) and the fetuses with deteriorated parameters (cases A-D) (Tables 2–10).

**Table 2:** Comparison of MCP-1 protein concentration in fetal plasma measured with ELISA between the case E and the others.

	0 H	4 H	8 H	12 H
Cases A-D	0.0 [9.7]	27.4 [17.4]	13.1 [22.9]	22.1 [22.1]
Case E	0.0	13.1	25.1	28.1

All the units for the presented values are pg/mL. All presented values for cases A-D are the group median [IQR].

**Table 3:** Comparison of TNF-α protein concentration in fetal plasma measured with ELISA between the case E and the others.

	0 H	4 H	8 H	12 H
Cases A-D	82.8 [43.0]	65.2 [38.9]	38.2 [39.9]	9.3 [36.8]
Case E	73.8	174.8	132.5	182.6

All the units for the presented values are pg/mL. All presented values for cases A-D are the group median [IQR].

**Table 4:** Comparison of relative expression of inflammatory cytokine/chemokine mRNA levels in fetal circulating immunocytes immediately after induction of EVE therapy measured with quantitative PCR between the case E and the others.

	IL-1β	TNF-α	IL-6	IL-8	MCP-2
Cases A-D	0.9 [0.4]	0.7 [0.4]	1.6 [0.3]	0.8 [0.0]	0.7 [1.2]
Case E	0.2	0.4	3.0	1.4	0.3

All the units for the presented values are normalised expression *vs*. the value for Control group. All presented values for cases A-D are the group median [IQR].

**Table 5:** Comparison of relative expression of inflammatory cytokine/chemokine mRNA levels in fetal circulating immunocytes 12 hours after induction of EVE therapy measured with quantitative PCR between the case E and the others.

	IL-1β	TNF-α	IL-6	IL-8	MCP-2
Cases A-D	1.6 [2.5]	1.2 [0.8]	3.5 [2.8]	1.0 [0.3]	37.7 [100.5]
Case E	14.2	2.8	5.2	2.1	35.1

All the units for the presented values are normalised expression *vs*. the value for Control group. All presented values for cases A-D are the group median [IQR].

**Table 6:** Comparison of relative expression of fetal liver acute phase protein mRNA levels measured with quantitative PCR between the case E and the others.

	CRP	SAA3	HEP
Cases A-D	10.1 [6.4]	50.6 [103.2]	1.6 [0.4]
Case E	2.3	84.6	0.5

All the units for the presented values are normalised expression *vs*. the value for Control group. All presented values for cases A-D are the group median [IQR].

**Table 7:** Comparison of relative expression of fetal pulmonary inflammatory cytokine/chemokine mRNA levels measured with quantitative PCR between the case E and the others.

	IL-1β	TNF-α	IL-6	IL-8	MCP-2
Cases A-D	1.8 [1.1]	1.1 [0.7]	4.9 [3.4]	3.6 [2.5]	7.7 [9.1]
Case E	1.6	1.8	2.0	2.6	48.8

All the units for the presented values are normalised expression *vs*. the value for Control group. All presented values for cases A-D are the group median [IQR].

**Table 8:** Comparison of relative expression of fetal brain inflammatory cytokine/chemokine mRNA levels measured with quantitative PCR between the case E and the others.

	IL-1β	TNF-α	IL-6	IL-8	MCP-2
Cases A-D	0.5 [0.5]	0.9 [0.3]	1.9 [3.5]	2.8 [1.8]	0.4 [1.6]
Case E	0.4	0.2	2.3	7.8	15.2

All the units for the presented values are normalised expression *vs*. the value for Control group. All presented values for cases A-D are the group median [IQR].

**Table 9:** Comparison of relative expression of fetal skin inflammatory cytokine/chemokine mRNA levels measured with quantitative PCR between the case E and the others.

	IL-1β	TNF-α	IL-6	IL-8	MCP-2
Cases A-D	1.6 [1.2]	0.7 [0.2]	1.5 [6.6]	3.1 [5.1]	3.1 [5.3]
Case E	8.1	1.8	1.0	10.8	3.6

All the units for the presented values are normalised expression *vs*. the value for Control group. All presented values for cases A-D are the group median [IQR].

**Table 10:** Comparison of qualitative scoring of histological inflammation in fetal airspace.

	Inflammatory score
Cases A-D	3.0 [0.0]
Case E	3.0 [0.8]

All presented values are the group median [IQR].

### Data relating to the assessment of pulmonary maturation

**Fig 8:**
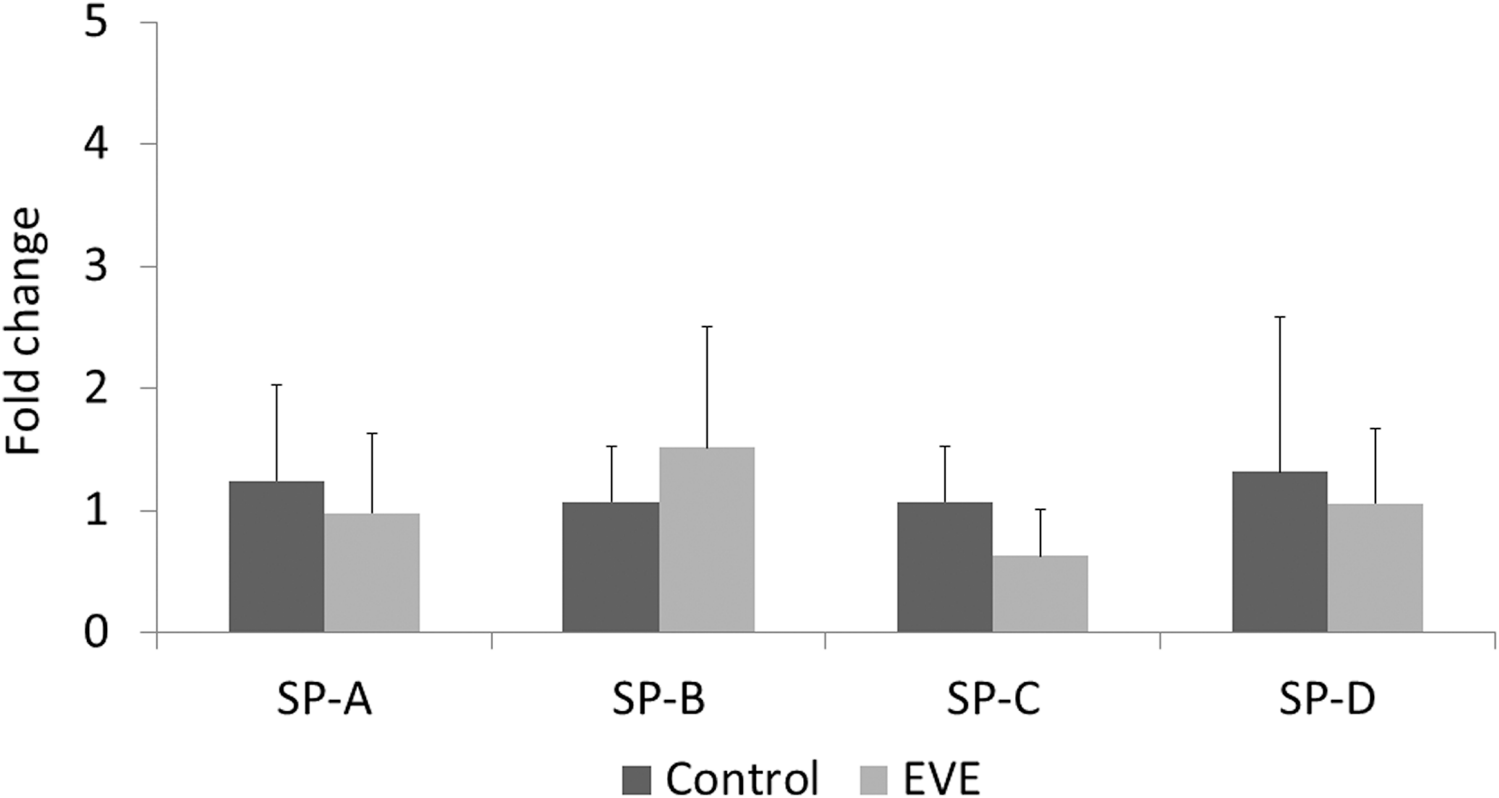
Relative expression of surfactant protein (SP) mRNA levels measured with quantitative PCR. All values are presented as bar charts with the group mean normalised expression *vs*. the value for Control group, with error bars representing SD. Significant difference was observed in no SP mRNA expression level between the two groups.

There was no significant difference in any SP mRNA expression levels in the fetal lung between the Control and the EVE groups (Fig 8).

## Discussion

In this study, we achieved limited fetal survival using EVE therapy. Mean survival time of the EVE group fetuses was 27.0 hours (Table 1) and only two of five fetuses completed their pre-determined study period (Fig 2D and 2E). Despite completing 40 hours of EVE therapy, one fetus demonstrated continuous deterioration including an increase in blood lactate levels and a decrease in circuit blood flow (Fig 2D). As blood lactate levels are used as a marker for tissue hypoxia, it is considered that a low (approximately less than 2 mmol/L [20]) blood lactate level correlates with fetal well-being and an appropriate degree of central and peripheral oxygenation; conversely, elevated blood lactate levels are associated with insufficient oxygen delivery to the peripheral tissues. The two fetuses that suddenly and unexpectedly deteriorated (due to a circuit corruption and a fatal arrhythmia; Fig 2B and 2C, respectively) maintained stable physiological parameters within reference ranges, and fairly low blood lactate levels (1.37 and 0.99 mmol/L, respectively) until immediately before the sudden deterioration.

EVE therapy induced an acute fetal inflammatory response relative to Control group animals. We hypothesised that our EVE system would induce only modest inflammation when it was successfully functioning, and that qualitatively and quantitatively larger systemic inflammatory changes would become apparent in animals in which treatment was unsuccessful (i.e. in cases where EVE therapy was unable to maintain normal physiological parameters). In the present study, only one case (Fig 2E) completed the pre-determined study period with physiological parameters remaining stable and within the optimal target range. The other four cases were either euthanised prior to 40 hours or exhibited a continuous deterioration in wellbeing. Although the sample sizes are too small to allow for a formal statistical analysis of these sub-group data, it is interesting to note that there was no apparent difference in global inflammation between these animals (either intermittently collected blood samples or autopsy samples), irrespective of how well they adapted to EVE therapy (Tables 2–10). Thus, although these data need to be interpreted with caution, they suggest that fetal inflammatory status may not be predictive of fetal wellbeing in EVE therapy. This observation is contrary to our hypothesis that changes in fetal inflammatory status may serve as a useful means of assessing treatment success in real-time.

Our data showed both systemic (Figs 3 and 4) and local (Figs 5–7) inflammation in fetuses maintained on EVE therapy, while there was no significant difference in the number of WBC or liver injury markers between the Control and the EVE groups (Table 1). This may, however, be a function of experimental length and a longer study could result in the identification of additional differences.

Intrauterine inflammation is associated with precocious maturation of the fetal lung as described above [6,7]. However, in the present study, the inflammation identified in the EVE group fetuses was not intense enough to promote lung maturation. Relative to control, significant increases were not observed in any SP mRNA expression levels (Fig 8) as hypothesised, despite the fetal lung showed relatively stronger inflammatory responses compared to other tissues analysed (Fig 6). Significant infiltration and consolidation in fetal airspace also suggest the existence of certain degree of inflammatory response in the lung of the EVE group fetuses (Fig 7). Elevated IL-6 proteins in cord blood have been reported to be predictive of a reduced risk of RDS, and have been shown to promote SP-A mRNA / protein expression in a time- and dose-dependent manner [21]. Although significant elevations in IL-6 mRNA expression levels in fetal blood immunocytes were not observed either acutely (0 hour) or after stabilisation (12 hours) on EVE therapy (Fig 4), statistically significant elevations in IL-6 mRNA expression levels were observed in the fetal lung (Fig 6). Given these results, the fact that there was no significant SP-A mRNA promotion in EVE group animals suggests that the inflammation brought by EVE therapy may be fairly mild and may not be clinically meaningful, although statistically significant inflammatory responses in several tissues were shown as discussed above.

The expression of CRP and SAA3 mRNA in the fetal liver was significantly elevated in the EVE group compared to the Control group (Fig 5). Increases were identified in the four fetuses with compromised physiological parameters as well as the single fetus that completed its protocol in good condition (Table 6). Theoretically, neutrophils activated by the contact with our EVE system could promote the synthesis of IL-1β, TNF-α, and IL-6 [11,22]; and those cytokines regulate the synthesis of CRP and SAA3 in the liver [23–25]. Interestingly, no significant elevations in IL-1β, TNF-α, or IL-6 mRNA levels were detected in RNA isolated from fetal blood immunocytes (Fig 4). Similar inconsistencies were also observed in RNA isolated from fetal lung and brain (Fig 6). These data suggest that the inflammatory mediators responsible for driving systemic responses are generated in the fetal tissues, or by tissue-resident immunocytes rather than by circulating immunocyte populations.

### Limitations of the study

The major limitation of this study is the low number of animals included, that impedes to draw robust conclusions, as this study required a significant investment of manpower and materiel.

Another limitation is the lack of the microbiological analysis. It might be possible that the inflammatory responses described above were induced by amniotic fluid infection, although fetuses were treated with antibiotics and the amniotic fluid was constantly UV filtered. Future studies will seek to address this uncertainty by culturing artificial amniotic fluid and fetal arterial blood over time and fetal tissues (lung and skin tissue) at autopsy.

It would also be of interest, in future studies, to determine whether or not fetal sex has an effect on EVE therapy outcomes.

### Strengths of the study

Although it has been reported that extracorporeal life support brings about inflammatory responses, the available data is still limited. Besides, inflammation intensity is altered by the way of blood access [14]. This is, to our knowledge, the first study to analyse the inflammatory response of the fetuses that were attached with A-V extracorporeal life support system using umbilical vessels. This study provides additional knowledge for further development of extracorporeal life support system including EVE therapy.

## Conclusion

In conclusion, limited survival of the fetus was achieved with EVE therapy. However, EVE therapy induced limited fetal inflammation and did not promote lung maturation. In the present study, fetal inflammatory status was not a meaningful predictor of fetal wellbeing. In contrast, sustained increases in fetal lactate levels may be predictive of impending fetal demise. These data provide additional insight into markers of treatment efficacy for the assessment of future studies.
